# Equitable modelling of brain imaging by counterfactual augmentation with morphologically constrained 3D deep generative models

**DOI:** 10.1016/j.media.2022.102723

**Published:** 2023-02

**Authors:** Guilherme Pombo, Robert Gray, M. Jorge Cardoso, Sebastien Ourselin, Geraint Rees, John Ashburner, Parashkev Nachev

**Affiliations:** a UCL Queen Square Institute of Neurology, University College London, London, UK; b School of Biomedical Engineering & Imaging Sciences, King’s College London, London, UK

**Keywords:** Counterfactuals, Deep generative models, Diffeomorphic deformations, Discriminative models, Data augmentation, Fairness, Equity, Brain imaging

## Abstract

We describe CounterSynth, a conditional generative model of diffeomorphic deformations that induce label-driven, biologically plausible changes in volumetric brain images. The model is intended to synthesise counterfactual training data augmentations for downstream discriminative modelling tasks where fidelity is limited by data imbalance, distributional instability, confounding, or underspecification, and exhibits inequitable performance across distinct subpopulations.

Focusing on demographic attributes, we evaluate the quality of synthesised counterfactuals with voxel-based morphometry, classification and regression of the conditioning attributes, and the Fréchet inception distance. Examining downstream discriminative performance in the context of engineered demographic imbalance and confounding, we use UK Biobank and OASIS magnetic resonance imaging data to benchmark CounterSynth augmentation against current solutions to these problems. We achieve state-of-the-art improvements, both in overall fidelity and equity. The source code for CounterSynth is available at https://github.com/guilherme-pombo/CounterSynth.

## Highlights

• 3D conditional generative model that generates brain demographic counterfactuals.
• Uses diffeomorphisms to modify only select morphological features of the brain.
• Counterfactuals mitigate the impact of demographic imbalance and spurious correlations.
• State-of-the-art improvements to overall fidelity and equity across subpopulations.

## Graphical abstract

**Figure:**
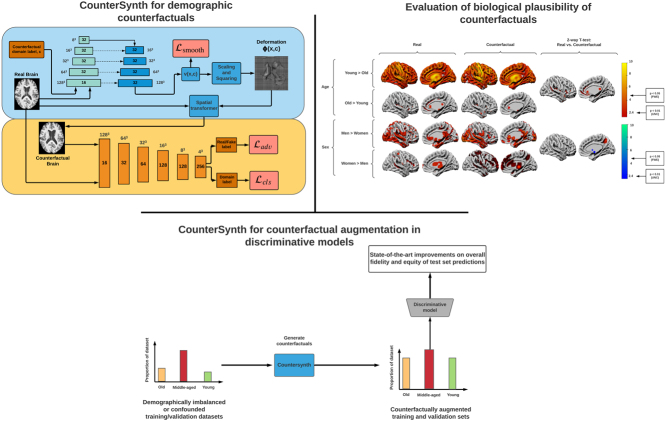

## 1 Introduction

The manifestations of neurological disease in the imaged brain are complex, reflecting the intersection of pathological, biological and instrumental forms of variation. A signal of interest here must typically be disentangled from a rich, widely distributed network of interacting factors: some irrelevant, others modulating. This problem is traditionally approached by assuming an *a priori*-defined, simple underlying compositionality – into discrete anatomical regions or continuous stereotactic spaces – that enables compact models to be deployed in a regional or voxel-wise manner (Mechelli et al., 2005, Cuingnet et al., 2012).

So strong a simplifying assumption places a hard limit on the complexity of the signals that can be modelled, but is inevitable where the scale of available data is small and the controllable flexibility of the models fitted to it low.

The revolutionary impact of deep learning on image modelling (Krizhevsky et al., 2012, He et al., 2016, Mnih et al., 2015) may enable us to relax this assumption (Liu et al., 2018, Li et al., 2014, Pombo et al., 2019, Havaei et al., 2017). Given sufficiently informative data, a deep neural network can implicitly find a decomposition of the image that best supports the task it is deployed to solve – prediction, prescription, or inference – trained end-to-end, guided by only weak inductive bias. Though attainable model expressivity is thereby enhanced, it falls on the data to control it. Crucially, any model here must rely on the data to distinguish between the target, foreground **signal**, and incidental, background **context** in which it is embedded.

Simple forms of context independence, such as invariance to translations (LeCun et al., 1989) and rotations (Cohen et al., 2018), or approximate viewpoint invariance (Sabour et al., 2017) can be incorporated in the model’s design. Equally, approximate invariance to geometric and intensity transformations can be promoted with on-the-fly data augmentation: this is how most deep image classifiers learn invariance to, for example, (small) affine and elastic transformations (Buslaev et al., 2020, Shorten and Khoshgoftaar, 2019) and – in the context of medical imaging – bias field and motion artefacts (MONAI, 2020). Models can even learn which augmentations to learn (Benton et al., 2020).

Nonetheless, where the context of a predictive signal is *itself* complex – for example, the age-related morphology of a brain in which small vessel disease is the target – no simple remedy is available. Retained sensitivity to context here not only impairs fidelity, it introduces vulnerability to distributional shifts, and may inject bias through irrelevant natural (confounder) (Buolamwini and Gebru, 2018, Hashimoto et al., 2018, Larrazabal et al., 2020) or sampling (collider) correlations (Griffith et al., 2020, Watson et al., 2019). The class imbalance and inadequate data representation common in the clinical domain (Loree et al., 2019, D’Amour et al., 2020) can only amplify the risks.

These concerns are far from merely hypothetical. Small vessel disease and age-related involutional change are closely correlated yet causally distinct (Arntz et al., 2016). A naive model could easily learn to rely on age (Szegedy et al., 2013, Nalisnick et al., 2018) to support an inference on small vessel disease, resulting in impaired performance in the decision space of highest clinical significance: where the two are unusually decorrelated. Similarly, a model tasked with distinguishing ischaemic from inflammatory causes of white matter hyperintensity (WMH) will be drawn into favouring the former in elderly men and the latter in young women (Spychala et al., 2017, Bonkhoff et al., 2021), reflecting the marked interactions of age and sex in the underlying patterns of disease prevalence. Many other examples are easy to adduce (Rao et al., 2017, Adeli et al., 2018, Yang et al., 2020, Larrazabal et al., 2020): the entanglement of pathological signals of interest with background, contextual factors is here not the exception, but the norm.

These concerns are also ethical. Amongst the many contextual factors in play are those – such as age, sex and ethnicity – that define demographic subpopulations. The performance of any model used in clinical care ought to be as close to invariant across all subpopulations as the available data allow. Such model *equity* may be defined as the extent of departure from the maximum achievable fidelity across identifiable subpopulations. An equitable model approaches the maximum achievable fidelity equally closely across all patients, regardless of their subpopulation identity (Carruthers et al., 2022). This broader notion of fidelity, extending beyond performance metrics drawn from the population as a whole, is inherent in the fundamental nature of medicine. Contextual invariance here must not only be implicitly promoted but explicitly demonstrated.

The space of possible solutions to this cardinal problem is dominated by two distinct approaches. One is to redistribute the model’s attention in training, through targeted data weighting or resampling (Khosla et al., 2012, Byrd and Lipton, 2019), context-dependent modulation of the objective (Sagawa et al., 2020), or adversarial mechanisms (Zhao et al., 2020). The redistributive nature of these approaches tends to incur a penalty on model fidelity, even if generalisation or equity may be improved (Goel et al., 2020), and the improvement seen in the context of the distribution shifts commonly present in clinical datasets is minor (Taori et al., 2020).

The alternative is to augment the training data with samples from a generative model expressive enough to capture the interactions between the target signal and its context (Choi et al., 2018, Choi et al., 2020, Zhu et al., 2017), in direct evolution of the use of generative models to expand minority classes synthetically (Goel et al., 2020, Mullick et al., 2019, Shamsolmoali et al., 2020). Though this approach is theoretically superior, its success is premised precisely on the disentanglement we are using it to promote, for the quality of the conditioned samples depends on the model’s knowledge of the conditioning feature. Moreover, a generative model ignorant of a target feature will tend to reproduce it poorly when tasked with generating a contextually modified counterfactual, often substituting non-pathological signal within areas outside, or in the tails of, the learnt distribution (Baur et al., 2021).

Here we propose to use conditional generative models of diffeomorphic spatial deformations (Ashburner, 2007, Blaiotta et al., 2018, Krebs et al., 2018, Dalca et al., 2018, Dorta et al., 2020), exploiting the expressive power of generative models to replicate contextual factors while limiting their propensity to interfere with target signals. Constraining the synthesis of an image to the spatial deformation of another provides the flexibility to capture common background morphological patterns of contextual modulation (Brickman et al., 2007, Baldinger-Melich et al., 2020), while leaving the brain identity and target pathological signals comparatively intact. This is so because a heavily regularised deformation field does not directly change signal intensities but displaces them, typically minimally. Deformation fields can be synthesised and re-sampled quickly, enabling on-the-fly augmentation even at very high image resolutions. Moreover, a model of deformations alone can have fewer parameters, and can therefore be easier to fit in the limited data regimes common in brain imaging. The limited availability of labelled data has inhibited development of fully volumetric unpaired image-to-image translation. Here we present the first framework for volumetric unpaired image-to-image translation in brain imaging.

Our general solution to the problem of promoting contextual invariance in models of brain imaging, *improving both model fidelity and equity*, is summarised as follows:

• We describe CounterSynth, the first 3D generative model capable of volumetric unpaired image-to-image translation. The model learns to synthesise counterfactual volumetric brain imaging for targeted, biologically informed augmentation of downstream discriminative models. Synthesis involves sampling a diffeomorphic deformation field conditioned on an original image and a contextual variable of interest, such as age or sex.
• The deformations modify only select morphological features of the source volume, presenting the target pathological signal against alternative, prescribed, counterfactually defined backgrounds. The deformations are easily regularised to promote minimal, biologically plausible deformations, even when conditioning on abnormal images. Modelling shape, but not signal, further enhances the robustness of the generative model to natural variations in signal intensity.
• CounterSynth is fast and memory efficient: it can generate training augmentations at sub-millimeter resolutions on-the-fly, even on consumer-grade hardware. The deformations can be resampled with negligible cost for fast synthesis at multiple resolutions.
• We use synthesised counterfactuals to mitigate the impact of demographic imbalance, spurious correlations, and collider bias on a range of brain imaging classification and regression tasks.
• We quantify the value – to both overall fidelity and equity – of augmenting data with synthesised counterfactuals. We compare with other GAN data augmentation methods (see Section 2.8), with confounder-free networks (see Section 1.1.4) and in comparison and combination with group distributionally robust optimisation (see Section 1.1.1), demonstrating superiority to current practice on all counts. In the course of this evaluation we introduce novel indices of equitable model performance and its cost.
• Our code is available at https://github.com/guilherme-pombo/CounterSynth.

### 1.1 Related work

An image classifier is a function that assigns each observation in image-space, X, a label in label-space, Y. Suppose we are given a family, Θ, of image classifiers, a loss (risk) function ℓ:Θ×(X×Y)→R, and N image-label pairs $\left(x_{1},y_{1}\right),\ldots ,\left(x_{N},y_{N}\right)\in X\times Y$. The usual approach to model selection, which is based on empirical risk minimisation (ERM), is to find a classifier θ∈Θ that minimises the empirical loss (risk), $\frac{1}{N}\sum_{k=1}^{N}\ell \left(\theta ,\left(x_{k},y_{k}\right)\right)$. If the observations are not sufficiently homogeneous, however, then prioritising *average* performance can lead to important subgroups being underserved (Buolamwini and Gebru, 2018, Sagawa et al., 2020).

#### 1.1.1 Group distributionally robust optimisation

In distributionally robust optimisation (DRO) (Ben-Tal et al., 2013, Rahimian and Mehrotra, 2019), one aims to minimise the worst-case expected loss over an ‘uncertainty set’ of distributions. In the group DRO setting (Hu et al., 2018, Oren et al., 2019, Sagawa et al., 2020), this minimisation is simply over the (instantaneous) worst-performing group of examples. In the context of neural network optimisation, given training data already divided into groups, Sagawa et al. (2020) minimise this empirical worst-group risk while demonstrating the importance of simultaneously enhancing generalisability through greater regularisation. They achieve markedly improved worst-group test set accuracy over ERM-based approaches, with minimal reductions in average test set performance. We benchmark CounterSynth against, and in conjunction with, group DRO. In particular we show that we can improve worst-group performance without harming average performance.

#### 1.1.2 Data augmentation with generative models

It is well-established that augmenting training data with images synthesised by generative models, such as generative adversarial networks (GANs) (Goodfellow et al., 2014) and variational auto-encoders (Kingma and Welling, 2013), can improve the performance of discriminative models (Ganesan et al., 2019, Sandfort et al., 2019, Han et al., 2019). In the domain of brain imaging, Han et al. (2019) demonstrate that augmenting the training data of neural tumour detection models with synthesised 2D slices of brains with tumours improves performance. Augmentation with synthetic data has also been used to address imbalance in the representation of heterogeneous subgroups, resulting in more equitable predictive performance (Goel et al., 2020, Mullick et al., 2019, Shamsolmoali et al., 2020). Subgroup data augmentation results in more equitable model performances than those obtained with group DRO (Goel et al., 2020). However, these results require a generative model capable of realistic synthesis of the underrepresented subgroups. Our model, CounterSynth, uses diffeomorphic deformations to achieve this using minimal compute resources, minimal training data, and a framework designed to promote biological plausibility.

#### 1.1.3 Unpaired image-to-image translation and GANs

Generating a set of diverse and realistic counterfactual images in order to expand an under-represented subgroup requires image-to-image translation. The paucity of paired data, means that we focus on unpaired translation, whereby a given image is transferred to a new ‘domain’ by a conditional generative model.

The state of the art here, StarGAN (v1 Choi et al., 2018; v2 Choi et al., 2020), is a type of generative adversarial network (GAN), Goodfellow et al. (2014) and Zhu et al., 2017, Choi et al., 2018. These two-part neural networks comprise a ‘generator’, G - a neural network that maps a random vector to image space - and a ‘discriminator’, D - a neural binary classifier that distinguishes between training data and the generator’s output. Given the data distribution p(X) and a latent distribution p(Z), the models train simultaneously by playing the two-player minimax game

(1) $$\underset{G}{min}\;\;\underset{D}{max}E_{x∼p\left(X\right)}\log D\left(x\right)+E_{z∼p\left(Z\right)}\log\left(1-D\left(G\left(z\right)\right)\right).$$

Under various technical conditions (Goodfellow et al., 2014, Kodali et al., 2017, Mescheder et al., 2018) the distribution G(z), z∼p(Z), converges to p(X).

StarGAN is a *conditional* GAN in the sense that, instead of noise, the generator takes as input an image and one or more domain labels. Multiple domain labels enables simultaneous transfer between multiple domains (e.g. when modelling portraits, changing hair colour *and* facial expression). Training on multiple domains simultaneously also ensures feature *disentanglement*. For example, again when modelling portraits, adding spectacles while keeping age constant. Entangled features in the context of brain imaging are features that appear to change together given a particular dataset. Features that are otherwise disentangled, might appear strongly correlated on smaller datasets or in particular clinical settings — for example, a longitudinal study of small vessel disease will have elements of the pathology entangled with features associated with ageing.

At StarGAN’s core is a type of ‘cycle consistency loss’ (Zhu et al., 2017), an L1 penalty on the reconstruction error accumulated by transferring an image to a domain and back again. In terms of the joint distributions of images and labels, p(X,Y), and the marginal label distribution p(Y), their cycle loss is

(2) $$E_{\left(x,y\right)∼p\left(X,Y\right),y_{\text{new}}∼p\left(Y\right)}{\left\|x-G\left(G\left(x,y_{\text{new}}\right),y\right)\right\|}_{1}.$$

This loss encourages approximately invertible domain transfers that, when regularised, should be no more complex than necessary. Domain transfers are thereby encouraged to preserve the visual content of the original image.

#### 1.1.4 Confounder-free neural network

The confounder-free neural network (CF-Net) learning scheme (Zhao et al., 2020) is designed to discourage medical image prediction models from acquiring biases in the presence of confounders. A minimax-type adversarial objective (1) is used to promote approximate invariance of the predictor’s featurisation of the image data to the presence of a given confounder in the input. The method has been validated on several challenging real-world diagnosis prediction tasks, including prediction of human immunodeficiency virus status from brain imaging data. We compare CF-Net to models trained on CounterSynth synthetic counterfactuals in Section 3.2.

#### 1.1.5 Paired brain-to-brain translation

Brain imaging is often replicated across multiple acquisition types (Miller et al., 2016, LaMontagne et al., 2019, Petersen et al., 2010), time periods, and stages of disease progression (LaMontagne et al., 2019, Petersen et al., 2010). This permits modelling the same brain under different conditions, learning to predict the characteristics of unseen test data from within-subject commonalities. Conditional GAN frameworks have been shown to be well suited for this task. For example, 4D-DANI-Net (Ravi et al., 2021) learns from matched pairs of volumes to perform domain transfer and ageing on Alzheimer’s disease imaging. Work done in Jung et al. (2021) achieves very similar results by modelling the disease progression with residual masks instead. Yurt et al. (2022) has demonstrated high-quality MRI volumetric recovery from undersampled acquisitions at 1 mm resolution with a 3D GAN that decomposes volumetric mappings into task-optimally ordered cross-sectional mappings. Similarly, Lan et al. (2021) uses spectral normalisation and feature matching to obtain state-of-the-art contrast synthesis. Dalmaz et al. (2021) instead uses Transformer architectures (Vaswani et al., 2017) to tackle the task of paired contrast synthesis. Li et al. (2022) show that paired-imaging translation GANs can facilitate MRI denoising, an application of great potential clinical value. In Korkmaz et al. (2022) instead use Transformer architectures for the task of MRI denoising. Rusak et al. (2020) found that given paired Partial Volume maps and corresponding MRI scans, GANs can learn to synthesise brain imaging with accurate tissue borders from any given partial volume map. It is critical to observe that all these methods require paired imaging. Learning synthesis from unpaired imaging is a far more difficult task (Zhu et al., 2017).

#### 1.1.6 Unpaired brain synthesis

StarGAN v2 is trained and tested on images of 256 × 256 resolution; our (volumetric) imaging dimensionality is greater by a factor of 32 (see Section 2.6). Though 3D-StyleGAN (Hong et al., 2021) shows that GANs are capable of generating realistic volumetric data unconditionally at 4 times the dimensionality of the original, 64 × 64 × 64, its sampling failures (see p8 of Hong et al. (2021)) suggest a more structured approach to prediction, such as one based on diffeomorphic displacements, is appropriate.

One successful example of structured prediction is described by Xia et al. (2021), who simulate progressive ageing and evolving Alzheimer’s changes in 2D brain slices in terms of additive masks. By avoiding modelling the full slice they reduce the parameterisation of the model and thereby avoid overfitting in smaller-scale data regimes.

To overcome the computational burden of full volumetric synthesis, distant spatial correlations can be sacrificed by modelling only patches/subvolumes of the original image. A conditional super- resolution model that operates on 64 × 40 × 64 subvolumes is described in Wang et al. (2020). Similarly, the conditional model described in Lin et al. (2021) operates on 96 × 96 × 48 regions of interest.

Note that despite the vast amount of literature on the task of paired brain-to-brain translation presented in Section 1.1.5, there is no current literature tackling unpaired volumetric modelling of tasks such as contrast synthesis/transfer and age and disease progression. One of the main contributions of our work is to propose a framework that enables unpaired image-to-image translation in the style of Choi et al. (2018) and Zhu et al. (2017) to be performed fully volumetrically and in an accurate fashion given the label limitations commonly present in the domain of brain imaging.

#### 1.1.7 Equitable model performance

Medicine is concerned with minimising the difference, at the individual level, between ideal and achieved clinical outcomes. Since the optimal management of an individual patient is typically unknown, it must be inferred from the population. In the setting of population heterogeneity, the fidelity of such inference will tend to be systematically biased in proportion to the representation of any given subpopulation (Larrazabal et al., 2020, Buolamwini and Gebru, 2018, D’Amour et al., 2020). The problem of equity then arises as consistent variation in model performance across different subpopulations.

Equity can be promoted by data manipulation prior to modelling (Johnson and Khoshgoftaar, 2019), or by directly incorporating appropriate metrics into training objectives (Oren et al., 2019, Sagawa et al., 2020, Barocas et al., 2017). In rebalancing the model’s attention across the population, any benefit to a given subpopulation may incur an undesirable cost elsewhere (Sagawa et al., 2020).

In this paper, we therefore consider variations in model performance at both the local (subpopulation) level and global level, quantifying the improvements at a local level with regards to the changes to global performance (see Section 2.3).

## 2 Methods

We use StarGAN-based unpaired image-to-image style transfer to synthesise realistic counterfactual brain imaging in terms of diffeomorphic deformations. These are infinitely differentiable, invertible coordinate transformations with infinitely differentiable inverses (Ashburner, 2007, Ashburner and Ridgway, 2013, Blaiotta et al., 2018).

Our restriction to deformations has the benefits itemised in the introduction; regularised diffeomorphic displacements naturally produce simpler, invertible domain transfers, so the cycle loss (2) is no longer needed. This considerably reduces model run time,^1^ as well as the complexity of the training objective. We also forego the ‘style vectors’ that were introduced in StarGAN v2 Choi et al. (2020) to further simplify the training objective; hence our model is closest to StarGAN v1.

Brain atrophy has been modelled in terms of spatial deformations (da Silva et al., 2020) using paired brain images and their associated atrophy maps. Population-level – as opposed to individual-level – ageing in longitudinal data has also be modelled with spatial deformations (Sivera et al., 2019, Huizinga et al., 2018).

### 2.1 Learning diffeomorphic deformations

Methods for predicting diffeomorphic deformations with neural networks by using ‘spatial transformer layers’ (Jaderberg et al., 2007) are described independently by Krebs et al. (2018) and Dalca et al. (2018). In both cases a convolutional neural network (CNN) predicts a coordinate transformation $\phi :R^{3}\mapsto R^{3}$ that registers a given source volume onto a given target volume. The deformation ϕ is represented in terms of a stationary velocity field, v, a real parameter t∈[0,1] and the identity transformation Id, defined such that

(3) $$\frac{\partial \phi^{\left(t\right)}}{\partial t}=v\left(\phi^{\left(t\right)}\right),\;\phi^{\left(0\right)}=\text{Id}.$$

Integrating t over [0,1] or, equivalently, exponentiating v recovers the deformation: $\phi =\phi^{\left(1\right)}=\exp\left(v\right)$. Their CNNs predict v from the source and target images, and then integrate v by scaling and squaring (Arsigny et al., 2006, Ashburner, 2007, Moler and Loan, 2003) before finally applying ϕ to the source image.

Crucially, all of the spatial transformations in this network are implemented in terms of (sub)differentiable ‘spatial transformer layers’ (Jaderberg et al., 2007), so the entire model can be optimised end-to-end, simply using (sub)gradient descent.

In the following section we describe how we use this technique to predict deformations for counterfactual synthesis.

### 2.2 Counterfactual synthesis with deformations

Our training objective is based on that of StarGAN (Choi et al., 2018). Suppose we have a set of domain labels {0,…,N} and let U denote the discrete uniform distribution over this set.

Using the notation from Choi et al. (2018), we use a discriminator $D_{src}$ to classify images as training data or not training data. The main component of our objective is the ‘non-saturating’ (Goodfellow et al., 2014) alternative to (1), which is used to encourage *all* deformations to be realistic:

$$L_{adv}=E_{x∼p\left(X\right)}\left[\log D_{src}\left(x\right)-E_{c∼U}\log D_{src}\left(x\circ \phi \left(x,c\right)\right)\right].$$

We use a second discriminator $D_{cls}$ to predict the domain of an image, and we let $D_{cls}\left(c|x\right)$ represent the probability distribution over domain labels predicted by $D_{cls}$. In terms of the joint distribution of images and (true) domain labels, p(X,Y), we minimise the following with respect to $D_{cls}$,

$$L_{cls}^{real}=-E_{\left(x,c\right)∼P\left(X,Y\right)}\log D_{cls}\left(c|x\right).$$

Given an image x, let ${\overset{¯}{U}}_{x}$ denote the uniform distribution over its counterfactual domain labels. To learn to generate counterfactuals, we minimise the following with respect to ϕ,

(4) $$L_{cls}^{fake}=-E_{x∼P\left(X\right),c∼\overset{¯}{U_{x}}}\log D_{cls}\left(c|x\circ \phi \left(x,c\right)\right).$$

We smooth the velocity field v in ϕ(x,c)=expv as in Dalca et al. (2018), by using a diffusion regulariser on its spatial gradients: for each voxel (i,j,k),

(5) $$L_{\text{smooth }}=E_{\left(x,c\right)∼P\left(X,Y\right)}\underset{i,j,k}{\sum }{\left\|\nabla v\left(x,c\right)\left(i,j,k\right)\right\|}^{2}.$$

Finally, for more stable training we use $R_{1}$ regularisation (Choi et al. (2020); see also Section 4.1 of Mescheder et al. (2018)),

(6) $$R_{1}=E_{x∼P\left(X\right)}{\left\|\nabla D\left(x\right)\right\|}^{2}.$$

In summary, our objective is to minimise $L_{disc}$ with respect to $D_{src}$ and $D_{cls}$, while minimising $L_{gen}$ with respect to ϕ, where

$$L_{disc}=-L_{adv}+L_{cls}^{real}+R_{1},$$

$$L_{gen}=L_{adv}+L_{cls}^{fake}+L_{\text{smooth }}.$$

When our domain label is given by a *continuous* variable, for example when conditioning on age, the training details are the same except that U denotes the continuous uniform distribution and $D_{src}$ is a regression model.

**Fig. 1:**
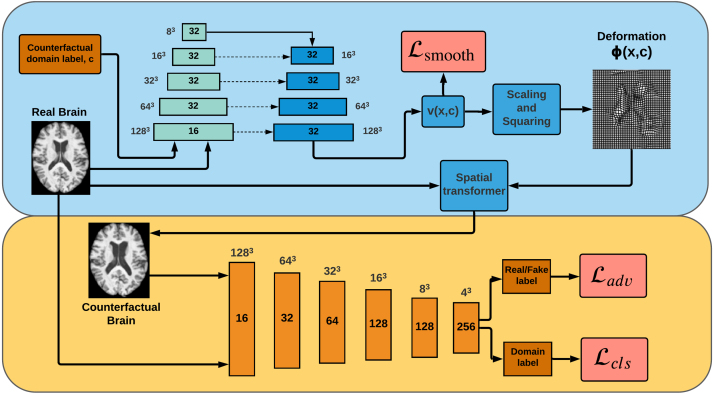
**Top**: The U-Net, plus scaling and squaring layers, for predicting and applying the deformation ϕ, via the velocity v. The input is the real image together with the counterfactual label added as a second image channel. Each block in both pyramids of U-Net layers is a convolutional layer that produces a feature map with 16, and thereafter 32, channels. Next to each block is its spatial resolution. These resolutions are decreased with max pooling and increased with nearest neighbour resampling. Dotted arrows represent skip connections. The scaling and squaring block is composed of spatial transformer layers. **Bottom**: The fully-convolutional discriminator for classifying real and synthesised images. Each upright block is a convolutional layer, producing feature maps with 16,…,256 channels. Above each block is the spatial resolution. Max pooling is used to reduce this resolution. Two probability distributions are predicted: real vs fake, and a distribution over domain labels.

### 2.3 Quantifying equity of performance

To quantify the impact of augmentation with counterfactual synthetic data on the relative equity of two classifiers (see Section 1.1.7), we would like a principled method of indexing variations in performance across the population. Econometrics provides an array of equality indices, such as the Gini index (Dorfman, 1979), the Theil index (Conceição and Ferreira, 2000) and the concentration index (Clarke and Van Ourti, 2010), largely based on statistical measures of dispersion.

In the context of model fidelity, equity can be trivially achieved by lowering global performance to that of the worst-performing subpopulation, but we typically wish to improve local performance without harming global performance. To capture the global impact of any *local* intervention, we need to measure *both* local and global effects.

First, we divide the population into the subpopulations $A_{1},\ldots ,A_{N}$, and denote by $a_{k}$ the mean performance of a given classifier on subpopulation $A_{k}$. Here we model global performance as the mean of these means: $\frac{1}{N}\sum_{k}a_{k}$. Of course, if each $A_{k}$ is equal in size this simplifies to the population mean. This approach weights every subpopulation equally, ensuring that its contribution does not depend on its size.

We use the individual $a_{k}$ to monitor local performance, paying particular attention to the worst-performing subgroup. Let $a_{1}^{\text{new}},\ldots ,a_{N}^{\text{new}}$ denote the subpopulation means based on the given model’s performance, and let $a_{1}^{\text{base}},\ldots ,a_{N}^{\text{base}}$ denote the subpopulation means based on the base model’s performance. We define the (normalised) global change in performance as

$$\Delta G=\frac{\underset{k}{\sum }\left(a_{k}^{\text{new}}-a_{k}^{base}\right)}{\underset{k}{\sum }a_{k}^{base}}.$$

Let $a_{p}^{\text{new}}$ be the subpopulation mean for the given classifier over its worst-performing subpopulation, and let $a_{q}^{\text{base}}$ be the subpopulation mean for the baseline classifier over its worst-performing subpopulation. Note the worst performing subpopulations can be different in the ‘base’ and ‘new’ cases. We define the (normalised) local change in performance as

$$\Delta L=\frac{a_{p}^{\text{new}}-a_{q}^{base}}{a_{q}^{base}}.$$

We form a simple, summary measure of the relative equity of a given model with respect to a baseline model from the mean of these two differentials. Since quantification of equity of performance is the aim of this index, we only invoke it when ΔL>0, for in the absence of any local improvement the global effects are moot. We refer to this as the Holistic Equity Index (HEI):

(7) $$HEI=\frac{\Delta G+\Delta L}{2}.$$

The HEI indexes the impact on the lowest performing subpopulation while taking into account the global cost across all subpopulations. Whenever we tabulate HEI in Section 3.2 the base model uses empirical risk minimisation (ERM); see Section 1.1.

### 2.4 CounterSynth training details

Our deformation generator is based on the architecture that was adapted by Dalca et al. (2018) for diffeomorphic registration from the U-Net (Çiçek et al., 2016); see Fig. 1. Our discriminators use the fully-convolutional model described in Isola et al. (2017), with the 2D convolutions replaced with 3D convolutions; see Fig. 1.

Whether conditioning on a discrete target domain label (e.g. self-reported male/female Biobank sex labels), or a continuous parameterisation of age, we replace the expectation over target domain labels in (4) with a single-sample Monte Carlo approximation, as in the original StarGAN framework.

For our experiments we shuffle the data then divide it into 80:10:10 training, validation and test splits. We train each model for 300 epochs, after which the model with the best performance on the validation set is selected. All tabulated metrics are computed on the test set.

The batch size was 128. We used the Adam optimiser (Kingma et al., 2014) with learning rate 10^−3^ for the generator and $2\times 10^{-4}$ for the discriminator (determined based on prior experience). $L^{2}$ weight regularisation was applied to all the non-bias parameters, with coefficient 10^−4^. The models were trained on an 8-card P100-based NVIDIA DGX-1.

We used stochastic discriminator augmentation (SDA) (Karras et al., 2020), which improves GAN performance in the absence of overwhelming amounts of training data. The augmentation functions were imported from MONAI v0.6 (MONAI, 2020) and applied to each training example independently with a probability of 0.8 (as recommended in Karras et al. (2020)). We used random affine and elastic deformations, nonlinear histogram transformations, contrast changes and additive Gaussian noise.

### 2.5 Predictive model training details

For the predictive tasks we use the official implementation of the current state of the art age and sex prediction model (Peng et al., 2021), which is implemented in PyTorch (Paszke et al., 2019). We used the Kingma et al. (2014) optimiser, with default settings, and a batch size of 128. We use the training data augmentation functions listed in Section 2.4, but with a lower probability of 0.2 (outside the SDA framework, high probabilities are not required).

All models are trained five times, for 200 epochs each, with different 80:7:13 training, validation and test splits (we increased the size of the test set at the expense of the validation set until it reached 2000 participants, to boost the numbers of under-represented demographics). To reiterate, the data used here does *not* overlap with the data used to train CounterSynth. All models are trained with oversampling of the minority classes and demographics. The model with the highest validation set balanced accuracy is then evaluated on the test set. All models were trained on an 8-card P100-based NVIDIA DGX-1.

Our experiments with DRO are based on empirical worst-group risk optimisation, see Section 1.1. The most effective version of this method requires large amounts of data set-specific $L^{2}$ regularisation (see Section 3.2 of Sagawa et al. (2020)). To find these values we performed a cross-validated grid-search using the training and validation sets. An $L^{2}$ regularisation coefficient of 0.01 provided the best results for all tasks, a finding which is consistent with the values used in Sagawa et al. (2020).

### 2.6 The data

We use two publicly available sets of brain magnetic resonance imaging (MRI) data: UK Biobank (Miller et al., 2016) and OASIS-3 (LaMontagne et al., 2019).

#### 2.6.1 UK Biobank

The UK Biobank (Miller et al., 2016) biomedical database contains a variety of brain magnetic resonance imaging (MRI) plus metadata (age, sex, etc.) from UK resident volunteers. From the T1-weighted brain imaging we randomly selected 30 K unique participants (ratio of men to women 54:46, mean age 52.7 years, standard deviation 7.5 years, range 38–80 years). We shuffled and then split the data into 15 K participants for training and testing CounterSynth, and a different 15 K participants for the remaining down-stream tasks.

To prevent our models relying only on linear differences in head volume and shape, all volumes were affine registered^2^ to MNI152 standard space (Collins et al., 1994) using SPM (Ashburner et al., 2005) and then cropped and down-sampled to 128 × 128 × 128 resolution. For the experiments in Section 3.2 we down-sampled the imaging further to 64 × 64 × 64 to facilitate multiple training runs.

For the counterfactual synthesis task (Section 3.1) we model age and sex, both of which are self-reported. They can be predicted with very high accuracy from brain imaging (Peng et al., 2021). For the predictive tasks (Section 3.2) we use age, sex and the total volume of white matter hyperintensities (WMH). The WMH data was derived automatically by using  (Griffanti et al., 2016) with subsequent quality control by the UK Biobank team (Alfaro-Almagro et al., 2018).

Wherever we use discrete domain labels, we bin age into ‘younger’ (age ∈[0,50]), ‘middle-aged’ (age ∈(51,55]) and ‘older’ (age ∈(55,80]) (these intervals are chosen to be roughly equal in size) and WMH volume into top quartile versus bottom three quartiles. Biobank’s sex variable is binary.

#### 2.6.2 OASIS-3

To evaluate the quality of our age counterfactual synthetics we use the OASIS-3 dataset (LaMontagne et al., 2019) as a ground truth. OASIS-3 is a longitudinal compilation of brain imaging data spanning 42 to 95 years of age. Participants include 609 cognitively normal adults, and 489 individuals at various stages of cognitive decline. For the purpose of evaluating only healthy ageing, we removed cognitively impaired individuals. We employ T1-weighted imaging only, and identically to UK Biobank, all data were affine registered to MNI152 standard space (Collins et al., 1994) using SPM (Ashburner et al., 2005) and then cropped and down-sampled to 128 × 128 × 128 resolution.

We use the first available scan of each participant as the brain volume from which the models predict the age counterfactual. The last available scan is used as the ground truth on which we evaluate the quality of the counterfactuals. The average elapsed time between image pairs is 4.3 years.

### 2.7 Evaluating quality of counterfactual synthesis

For small age increments, one can use longitudinal datasets such as OASIS-3 (LaMontagne et al., 2019) to evaluate objectively the accuracy of a model’s ageing process. We use this helpful evaluation benchmark in section Section 3.1.3, comparing the Structural Similarity Index (SSIM) (Wang et al., 2004, Renieblas et al., 2017), as well the mean average error (MAE), between the actual aged brain and the predicted counterfactual aged brain. For large age intervals, however, there exists no ground truth, and for other demographic attributes such as sex, counterfactual synthesis cannot be evaluated by a simple image comparison, for the synthetic image is definitionally inexistent.

We therefore quantify the fidelity of the conditioning biological signals – here age and sex – in three complementary ways. First, we use voxel-based brain morphometry (VBM) (Good et al., 2001) to compare their regional correlates across real and synthetic images. Second, we use a discriminative model trained exclusively on real data to compare their relative predictability. The former provides an index of the spatial fidelity of the counterfactual anatomy, the latter of its predictability from real data. Third, in the absence of likelihoods we use the Fréchet inception distance (FID) (Heusel et al., 2017), the current standard for quantifying the overall quality of GAN-generated image.

#### 2.7.1 Voxel-based morphometry (VBM)

VBM is conventionally used to infer the population-level anatomical correlates of a set of biological factors of interest (Mechelli et al., 2005, Ashburner and Friston, 2001). This is done via a mass-univariate voxel-wise comparison of tissue concentrations across homologous regions, enabled by prior non-linear registration to a common stereotactic space. Here we implemented this within SPM’s well-established pre-processing and statistical framework. This allows us to compare demographic-associated structural changes in real brain volumes with the structural changes obtained from counterfactual synthetics.

#### 2.7.2 Fréchet inception distance (FID)

To enable comparison with other likelihood-free generative models, we compute the Fréchet inception distance (FID) (Heusel et al., 2017) between our original data and the synthesised counterfactuals.

FID is computed from the hidden activations produced when these two sets of data are passed through an image model. We use the official FID implementation provided in Heusel et al. (2017), which we adapted to PyTorch. This is based on the widely-used Inception v3 model trained on Imagenet. In terms of the sample means $\mu_{orig.}$, $\mu_{gen.}$ and covariances $\Sigma_{orig.}$, $\Sigma_{gen.}$ of these sets of hidden activations, and in terms of the $L^{2}$ norm ${\left\|\cdot \right\|}_{2}$ and trace operator tr, the FID is defined as

(8) $${\left\|\mu_{orig.}-\mu_{gen.}\right\|}_{2}^{2}+\text{tr}\left(\Sigma_{orig.}+\Sigma_{gen.}-2\sqrt{\Sigma_{orig.}\Sigma_{gen.}}\right).$$

Since FID applies only to 2D images, we extracted a 128 × 128 slice along each axis in turn. We chose the slice with the maximum voxel-wise t-statistic for the relevant attribute (see Section 3.1.1).

### 2.8 Baseline methods for unpaired counterfactual synthesis

There are no unpaired methods in the literature for comparison with ours: we were therefore compelled to adapt other work to provide suitable baselines. The most promising candidate (Xia et al., 2021), uses a GAN and seeks to age a brain while preserving its identity. To achieve this, the authors use an ‘identity-preservation’ loss that encourages the image changes to be positively correlated with age change, as well as a self-reconstruction loss, which is designed to encourage smoother age-related changes. Furthermore, rather than synthesising a whole image, the framework synthesises only a mask, which is then added to the original brain image to simulate the process of ageing. The original implementation published by the authors operates only in 2D. We extend to 3D by replacing 2D convolutions in the latent feature extractor with 3D convolutions, as well as adding one extra layer to the Encoders and Decoders to make the increased dimensionality more manageable. We forego modelling the health state vector as it is not relevant in the context of our work. For the resolution of our experiments, in 2D, the final convolutional layer’s output dimensionality is 8×8×32=2048. In 3D, due to the extra layer of convolutions, it is 4×4×4×64=4096, so we increase the dimensionality of the fully-connected layers to accommodate this extra information. The age vectors are the same both in 2D and 3D. For simplicity we denote this method with the acronym **LGAN** throughout the rest of the paper.

For our second baseline, we adapt unconditional volumetric brain generation with StyleGANs (Hong et al., 2021) to enable image-to-image translation using (Richardson et al., 2021). This allows us to exploit the pretrained StyleGAN networks provided by the authors of Hong et al. (2021), specifically network ‘2 mm-fd64’. We need only train an encoder network that directly generates a series of style vectors which are then fed into the provided StyleGAN network. Unlike CounterSynth and Xia et al. (2021), this method does not operate with attribute vectors, and instead requires a target volume from which the style vectors are to be extracted. To evaluate it at test time, we therefore randomly select a brain image from the test set with the desired attribute we want to synthesise while keeping all the other attributes static. Note that the optimal manner of conducting such transfer is a research topic on its own, and is outside the scope of this paper. We denote this method **SGAN** throughout the rest of the text.

### 2.9 Summary of methods for equity improvement

We compare the following approaches based on ERM, group DRO (Section 1.1.1), confounder-free networks (Section 1.1.4) and CounterSynth training augmentation (Section 2.4). To avoid information leaks, whenever we use GAN-based augmentations we use two models, one trained exclusively to produce age counterfactuals, and one to produce sex counterfactuals. This ensures that the age counterfactuals do not carry over any sex information and vice-versa. The methods we baseline for correction of downstream predictive inequity in experiments section Section 3.2 are as follows:

• **ERM**: In line with empirical risk minimisation (see Section 1.1), we simply use online stochastic gradient descent (SGD) to optimise the predictive models.
• **DRO**: We use the official implementation of the group DRO optimiser (Algorithm 1 in Sagawa et al. (2020), which has a similar run time to SGD) with an $L^{2}$ regularisation coefficient determined by grid-searching (Section 3.2 of Sagawa et al. (2020)). In the group DRO setting one selects demographic attributes believed to be spuriously correlated with the target variable; see Section 2.1 of Sagawa et al. (2020). In the case of sex classification (Section 3.2.1) we choose age, in the case of WMH volume classification (Sections 3.2.3– 3.2.4) we choose age and sex.
• **ERM with counterfactuals (ERM**+**CSYNTH)**: We use the ERM approach above, but we augment the training (validation) set with counterfactuals synthesised by CounterSynth (CSYNTH for short) from the training (validation) set until the under-represented demographic is as numerous as the others. A new set of counterfactuals is synthesised at each epoch from equal numbers of the majority demographic. We adopt the same strategy with SGAN and LGAN augmentations as baselines, denoted (ERM + SGAN) and (ERM + LGAN) respectively
• **DRO**+**CSYNTH**, **DRO**+**SGAN**, **DRO**+**LGAN** : As above, but with the ERM approach replaced with the DRO approach.
• **CF-Net**: We use the official implementation of the Confounder-Free network (CF-Net), Zhao et al. (2020); see Section 1.1.4. CF-Net learns a featurisation of the data that is approximately invariant to a chosen demographic attribute. In the case of sex classification (Section 3.2.1) and WMH volume classification (Section 3.2.3) we choose age. The official implementation does not support regression objectives (Section 3.2.2) or tasks with multiple confounders (Section 3.2.4), so we omit comparisons with CF-Net in these cases. CF-Net also takes significantly longer to converge than the other baseline models: we therefore leave training it in conjunction with GAN data augmentation for future work.

## 3 Experiments and results

### 3.1 Counterfactual synthesis

In order to appropriately assess the quality of the synthesised counterfactual data we train and evaluate our CounterSynth and baseline models in two different scenarios:

1. We use the ‘younger’, ‘middle-aged’ and ‘older’ age bins defined in Section 2.6, and separately we use Biobank’s self-reported (binary) sex metadata. The models trained under this regime will be used for the data augmentation in the downstream predictive equity experiments in 3.2, as well for the evaluation of counterfactual quality when there are no ground truths Sections 3.1.1, 3.1.2 and 3.1.4.
2. We train the models with continuous age values, so that we can evaluate them on longitudinal ground truths using the dataset defined in Section 2.6.2.

In Fig. 3, Fig. 4 we present some example counterfactual synthesis for Biobank brains.

#### 3.1.1 Experiment: voxel-based morphometry

We used SPM’s unified tissue segmentation and normalisation algorithm (Ashburner et al., 2005) to generate non-linearly registered grey matter segmentations of 1000 real and 1000 counterfactual images conditioned on age or sex, all drawn from the test set. At each voxel, grey matter concentration, the dependent variable, was entered into a multiple regression with age, sex, origin, and total intracranial volume as independent variables. After model estimation, two one-tailed t-tests were performed on the regression coefficients (slopes) of the age and sex variables, with the resulting SPMs thresholded at p<0.05 FWE (cluster-based family-wise correction), and p<0.01 (uncorrected cluster forming threshold). An unusually lenient uncorrected threshold was deliberately chosen to reveal inferred areas to their maximum extent. Two-tailed t-tests were performed on the coefficients of the origin variable – real or counterfactual – separately for the age and sex contrasts, with identical thresholding. Anatomical labels based on the AAL3 atlas (Rolls et al., 2020) were assigned to the peak of each cluster, and the top 10 regions were compared.

**Fig. 2:**
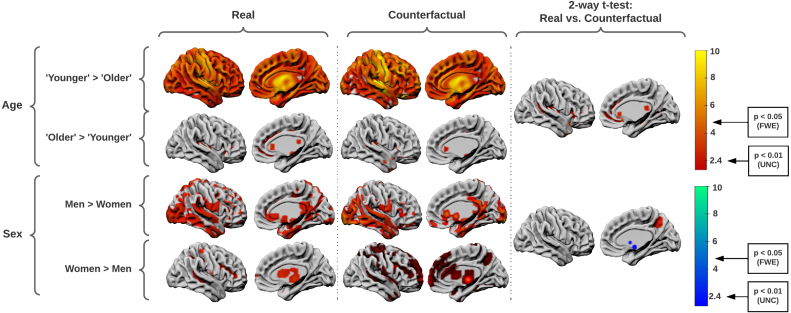
SPM’s VBM t-statistics for grey matter changes induced by the CounterSynth age and sex deformations. Leftmost, the grey matter changes associated with age and sex in the original data. Middle, those same changes but in the synthesised counterfactuals. Rightmost, the two one-tailed post-loc t-tests that show voxels where the real and counterfactual regression coefficients differ, the differences being negligible. There are two T-value thresholds to consider, the uncorrected estimation threshold for p<0.01 (UNC) and the family-wise estimation threshold for p<0.05 (FWE).

Inspection of the resultant maps (Fig. 2) shows similar anatomical patterns for all contrasts. For both age and sex, the CounterSynth VBM t-statistics matched 95% of the anatomical labels identified in the real data. Few regions in the counterfactual vs real comparison survived the extremely lenient uncorrected threshold.

Note the fidelity quantified here is of the conditioning, background signal, not the foreground signal we seek to preserve. This is quantified by the downstream discriminative model (see Section 3.2).

**Fig. 3:**
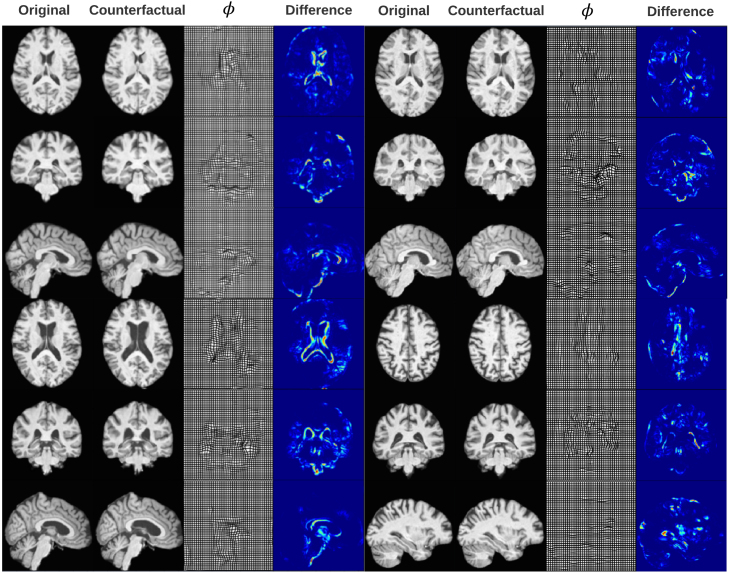
Example synthesis of volumetric counterfactuals for sex and discrete age bins tested on four different participants **Age**: The first four columns (from left to right) are age counterfactuals. The first three rows show a ‘middle-aged’ brain and its ‘younger’ counterfactual. The last three rows show the ‘older’ counterfactual for a ‘middle-aged’ brain; **Sex**: Columns five to eight show sex counterfactuals. The first three rows show a ‘female’ brain and its ‘male’ counterfactual. The second three rows show the ‘female’ counterfactual for a ‘male’ brain.

**Fig. 4:**
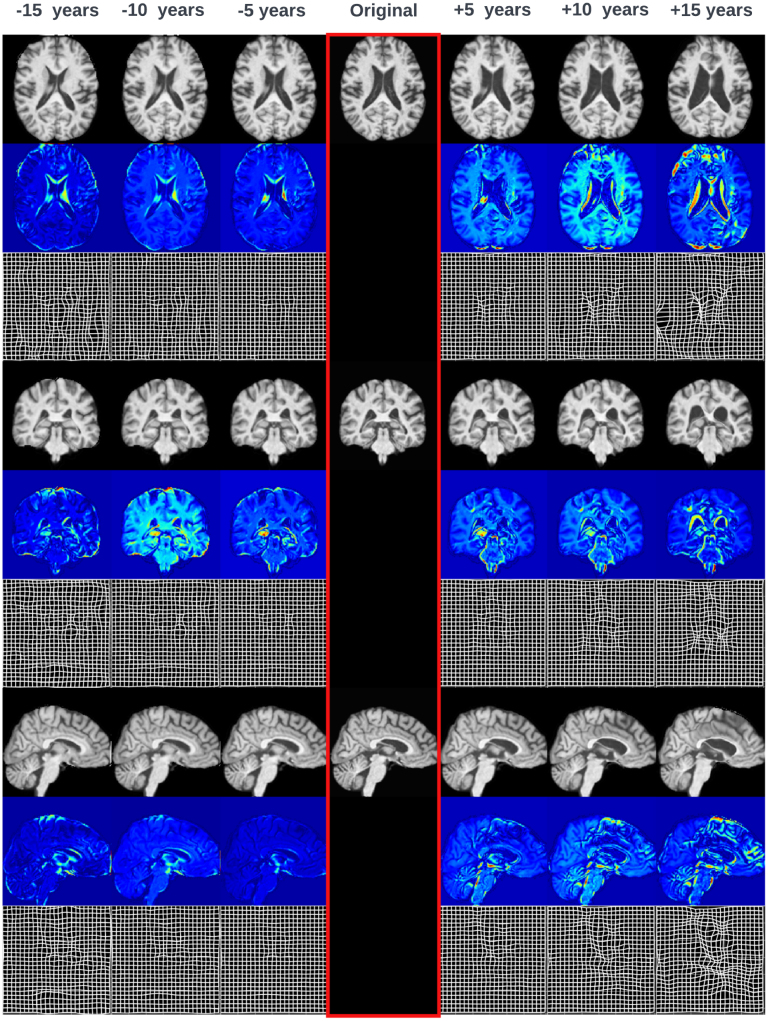
Example synthesis of continuous age counterfactuals for a single participant. To the left of the original we can see the brain being de-aged in 5 year increments and to the right the brain being aged in 5 year increments. Under each sagittal, coronal and axial slices we show the absolute difference maps between the counterfactual slice and the original one, as well as the displacement fields associated with each transformation. Ageing transformations enlarge the lateral ventricles and expand the size of the sulci. Deageing transformations produce tightened lateral ventricles and sulci. These morphological changes are inline with the ageing deformations described in literature (Sivera et al., 2019, Huizinga et al., 2018).

#### 3.1.2 Experiment: Fréchet inception distance

Employing the FID metric defined in Section 2.7.2 we evaluate the quality of each method, using 1000 test volumes from the UK Biobank dataset, and all possible attribute transfer combinations. This produces three sets of counterfactual examples from the test set:

1. We replace each brain with its sex counterfactual.
2. We use the three discrete age classes defined in Section 2.6.1 and for each brain we replace it with the corresponding two age counterfactuals (e.g. if it is a ‘younger’ brain we replace it with ‘middle-aged’ and ‘older’ counterfactuals).
3. We apply an image transformation twice in succession: first replacing each brain with its sex counterfactual, then replacing this counterfactual with its two age counterfactuals.

The resulting FID scores are presented in Table 1. Lower values correspond to greater visual (metrical and perceptual) similarity. CounterSynth’s low FIDs reflect the propensity of regularised deformations to leave much of the original image essentially unchanged: a key part of our motivation for using them. Since LGAN produces only a mask added to the original brain volume, its FID scores are also low, though four times higher than CounterSynth’s. SGAN, which must generate the entire brain volume and is therefore faced with the hardest task has the highest FID scores (consistent with the values presented in the original paper for unconditional brain generation Hong et al., 2021). For all methods, the more attributes are transferred, the higher the FID scores, as the modelling task inevitably involves more extensive modification.

**Table 1:** Average Fréchet inception distances between original data and synthetic data.

Model	Axial	Coronal	Sagittal
CounterSynth (age)	**11.9**	**10.7**	**9.5**
CounterSynth (sex)	**9.8**	**9.9**	**9.1**
CounterSynth (age & sex)	**12.4**	**11.1**	**9.8**
SGAN (age)	71.3	88.5	106.9
SGAN (sex)	78.4	84.2	98.7
SGAN (age & sex)	82.3	89.8	111.3
LGAN (age)	46.7	47.3	39.6
LGAN (sex)	42.5	43.8	33.9
LGAN (age & sex)	51.7	52.4	47.6

#### 3.1.3 Experiment: age prediction

**Fig. 5:**
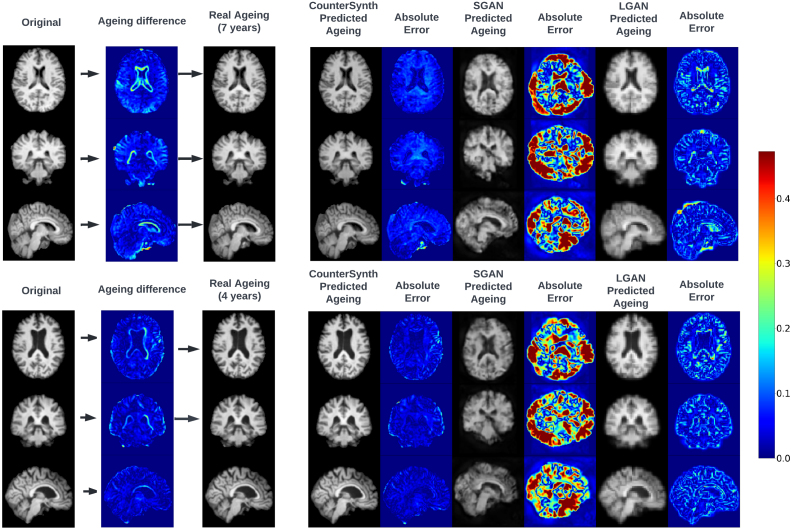
Real and predicted ageing for two participants from the OASIS-3 dataset. We present imaging of the participant’s brain at the first collect time point, followed by that same participant’s imaged brain at the final time point, along with associated absolute difference between the two images. Then for each method, we show the predicted brain image for the elapsed time frame (7 and 4 years) alongside with the absolute error between the predicted volume and the ground truth. For easier visual interpretation only the top 50th percentile of the error is shown.

**Fig. 6:**
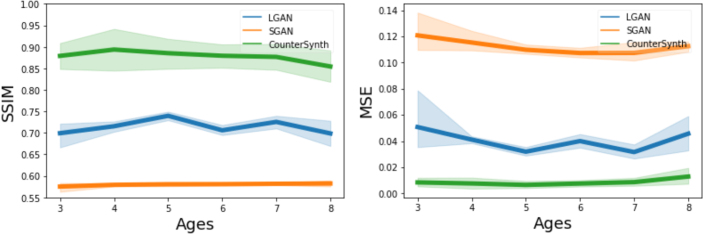
SSIM (higher is better) and MSE (lower is better) between the real aged brain and the predicted synthesised aged brain for varying amounts of ageing.

Using the dataset described in 2.6.2 we evaluate each model’s ability to predict the ageing process for a particular input brain volume. An example brain ageing prediction for each model is shown in Fig. 5. As the OASIS-3 dataset was unseen by any of our models, we perform a dataset-wide evaluation of the quality of the counterfactuals and present the results in Fig. 6.

Inspection of Fig. 5 and the results presented in Fig. 6 shows that the SGAN method is unsuited to counterfactual synthesis. The images look qualitatively similar to those in the published paper (Hong et al., 2021), and though they capture some of the desired attributes of the target image, their quality is too low to conclude whether or not the brain has been accurately aged. Furthermore, SGAN produces undesirable artefact intensities (white blur), outside of the area of the brain, which can be seen in Fig. 5, further increasing the error of its predictions. This is reflected in low SSIM and high MSE scores. LGAN counterfactuals closely resemble the ground truth, and exhibit some of the qualitative morphological changes associated with natural ageing. However, image artefacts corrupt important features such as sulcal configurations, resulting in comparatively low SSIMs. LGAN consistently produces blurry images which was reflected in a stubbornly high self-reconstruction loss. We hypothesise this is due to lack of either model capacity or data. We experimented with increasing LGAN’s model capacity revealed, which did result in higher fidelity reconstructions, but counterfactual sample quality deteriorated markedly. CounterSynth produces qualitatively the best images, yielding the highest SSIM scores across all three methods. Both in Fig. 5, Fig. 6 it is shown that the error of predictions goes up, as the amount of ageing to synthesise increases. This is to be expected as the number of external factors that effect the ageing of brain increases the more years pass. Note that OASIS-3 is entirely different from the data distribution CounterSynth was trained on: these results therefore demonstrate well the model’s ability to transfer intelligence from large-data to smaller-data regimes.

#### 3.1.4 Experiment: sex prediction and disentanglement

To quantify the fidelity of sex counterfactual synthesis, as well as its disentanglement from ageing, we trained sex classifiers, using the architecture described in Peng et al. (2021), to baseline accuracy on real data and compared their accuracy on counterfactual data. The models were trained once using the training participants set aside for the predictive modelling (see Section 2.6), attaining a test set accuracy of 99.2%. We then created three sets of counterfactual examples for each method as described in Section 3.1.2.

**Fig. 7:**
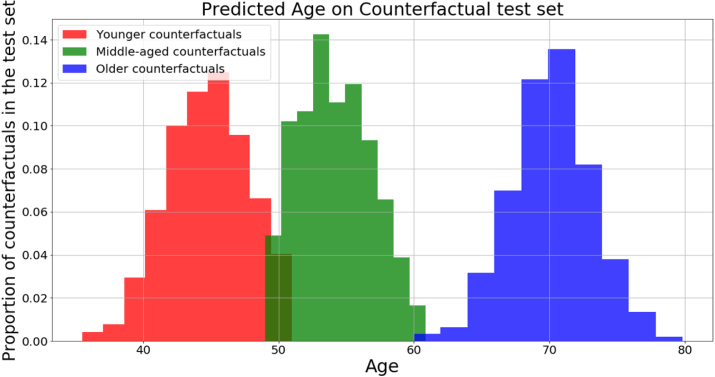
The distribution of predicted ages for the CounterSynth synthesised counterfactuals. **Red**: Distribution of predicted ages for the ‘middle-aged’ & ‘older’ participants transformed into ‘younger’ participants; **Green**: Distribution of predicted ages for the ‘younger’ & ‘older’ participants transformed into ‘middle-aged’ participants; **black**: Distribution of predicted ages for the ‘younger’ & ‘middle-aged’ participants transformed into ‘older’ participants.

The results presented in Table 2 show that SGAN modifies sex-related characteristics even when designed to change only age. This may be due to the artefacts generated by this method, as noted in Section 3.1.3. SGAN struggles to convey sex, perhaps owing to the subtlety of sex-related dimorphisms in the brain. Both LGAN and CounterSynth show levels of disentanglement of age and sex expected from the StarGAN framework, with CounterSynth results being superior. Taken together with the VBM maps presented in Fig. 2 this indicates that CounterSynth is preserving biological signals well during attribute transfer. To give a sense of the spread of counterfactual ages when using categorical buckets as opposed to continuous age differentials, we predicted the continuous ages of the counterfactuals in set (2) produced by CounterSynth. The histogram of predictions is shown in Fig. 7. It shows that range of ages produced for the categorical age buckets is fairly wide (average 10 years). This could be seen as an indication that smaller age buckets are required to get more precise age generation. However, in the context of their use for data augmentation, there is a trade-off between counterfactual age precision and the total computational budget required to train a model with counterfactual augmentation. We show in the following sections, that our age quantisation is sufficient to provide discriminative models with state-of-the-art performance on underrepresented age groups, despite the relatively course age ranges.

**Table 2:** Sex classification accuracy for counterfactual images produced by various methods.

Model	Age accuracy	Sex accuracy	Age & sex accuracy
CounterSynth	**97.6%**	**96.8%**	**96.4%**
SGAN	86.3%	74.9%	71.3%
LGAN	94.2%	85.5%	83.2%

### 3.2 Downstream predictive equity

In this section, we demonstrate CounterSynth’s ability to improve the average performance, and the worst-demographic-subpopulation performance, of a classifier trained on demographically-imbalanced data. We also demonstrate CounterSynth’s ability to lessen the extent to which a classifier learns spurious correlations between demographic attributes and the target label.

#### 3.2.1 Experiment: sex classification

For this experiment we create training sets with different levels of missingness of a chosen demographic (older people), and use them to train a classifier to predict sex. We use the approaches described in Section 2.9 to counter the negative effects of the resultant demographic imbalance on average performance, and to boost worst-demographic-subpopulation performance, as measured by balanced accuracy, precision and recall.

To simulate the missingness we create sets with the maximum possible equal number of ‘younger’ and ‘middle-aged’ participants (see Section 2.6 for age ranges), then add ‘older’ participants until they constitute a given percentage of the total. The percentages are 0, 1, 10 and 25. There are equal numbers of men and women in the ‘younger’, ’middle-aged’ and ‘older’ subpopulations.

**Fig. 8:**
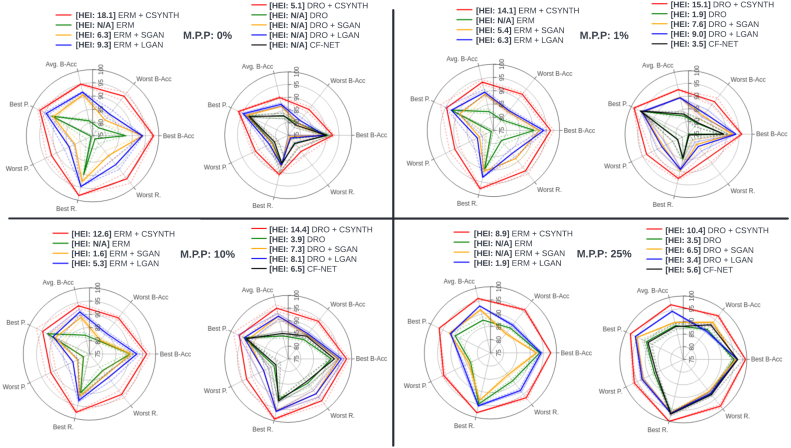
Spider plots depicting the performance of each model in terms of, on separate axes, average balanced accuracy (Avg B-Acc), best subpopulation balanced accuracy (Best B-Acc), worst subpopulation balanced accuracy (Worst B-Acc), best subpopulation precision (Best R.), worst subpopulation precision (Worst R), best subpopulation recall (Best R.), worst subpopulation recall (Worst R.) and, in the legend, the HEI score. The ideal model should be maximal along each axis, yielding an equilateral heptagon shape of maximum surface area, and should exhibit the largest HEI. Dotted lines indicate 1 standard deviation. The minority population percentage (M.P.P.) is manipulated across panels as indicated in the legend. Here we present test set results for sex classification with varying representations of ‘older’ participants. The number of ‘young’ and ‘middle-aged’ patients in the training and validation sets is 5153, 452 respectively. Of the ‘older’ participants in the training and validation sets respectively, 1% amounts to 52, 4 participants, 10% amounts to 572, 50 participants, and 25% amounts to 1717, 151 participants. Here ‘N/A’ indicates that ΔL≤0 (see Section 2.3), so the HEI does not apply.

For each combination of model and missingness we present in Fig. 8 balanced accuracy, precision and recall for the best- and worst-preforming demographics, average balanced accuracy, and our HEI index (7).

The spider plots presented in Fig. 8 show that ERM’s performance on the most under-represented subpopulation suffers a severe deterioration when in the setting of marked class imbalance. This is consistent with the findings presented in Oren et al., 2019, Sagawa et al., 2020 and Zhao et al., 2020, Buolamwini and Gebru, 2018. Only when the ‘older’ patients retained in the dataset reach 25% does the performance gap between the best and worst performing subpopulations begin to close. However, it is noticeable that even at this ratio there is a significant difference between the best performing subpopulation and the worst.

We find the performance of CF-Net and DRO without the use of any augmentation to be roughly equivalent. This is likely explained by their use of training objectives that promote invariance to features relevant to particular demographics. Compared with ERM, both methods consistently improve performance on the worst-off subpopulation, although only marginally when the subpopulation percentages are low (0% and 1%). One explanation for this failure at low subpopulation representations is that both DRO and CF-Net need the relevant under-represented subpopulation to be present in the data before they can learn which features to be invariant to.

Training with counterfactuals improves general performance regardless of the model that produces the augmentations. Most noticeable is the fact that the improvements come from better predictions on the worst-off population. This is visualised in the spider plots as an increase in the total area covered by each method relative to ERM. Larger HEI index are always accompanied with a larger area increase in the spider plot. Counterfactuals produced with CounterSynth result in the largest improvements. As shown in Sections 3.1.2, 3.1.3 the quality of CounterSynth counterfactuals is the highest, allowing for the best simulation of the missing subpopulation demographics, and thereby resulting in the highest improvements to the under-represented demographics. Note that at 25% representation, ERM + CSYNTH’s and DRO + CSYNTH’s performance plots are essentially equilateral.

#### 3.2.2 Experiment: age regression

For this experiment we create training sets with different sex imbalances, and use them to train an age prediction model.

We test the approaches described at the start of Section 3.2 to counter the negative effect on average performance and to boost worst-demographic-subpopulation performance, as measured by mean absolute error (MAE). We do not use group DRO because the official algorithm was unstable in conjunction with our regression objectives, and we do not use CF-Net because the official implementation does not support a regression objective. To simulate the missingness we create sets with the maximum possible number of female participants and equal numbers of ‘younger’, ‘middle-aged’ and ‘older’ participants; see Section 2.6. Then we add male participants until they constitute a given percentage of the total. The percentages are 0, 1, 10 and 25.

Table 3 shows that counterfactual augmentation drastically reduces the model’s error rate on the under-represented subpopulation, while also consistently improving its performance on the rest of the population. Similarly to the results shown in the previous section, CounterSynth augmentation leads to the biggest improvements in overall accuracy and, most importantly to us, the biggest reductions in error for the under-represented demographic. In this set of experiments, SGAN augmentations provide only a very small error attenuation in the male population, possibly because sex counterfactuals require more fine-grained volume changes and, as illustrated in Sections 3.1.2, 3.1.3 and 3.1.4, SGAN counterfactuals are too low-resolution to represent the subtleties of sex dimorphism. For similar reasons LGAN’s counterfactuals provide a smaller attenuation of the imbalance than CounterSynth.

**Table 3:** Mean absolute errors over the test set for age regression with varying representation of male participants. The number of women in the training and validation sets is 7627, 846 respectively. The number of men in the training and validation sets respectively for the different percentages are, for 2.5%, 195, 21; for 5%, 404, 44; for 10%, 846 and 94; for 25%, 2542, 282.

Method	Men MAE	Women MAE
ERM, 0%	5.14 ± 0.70	3.15 ± 0.44
**ERM + CSYNTH**, 0%	**3.89**± 0.40	**3.13**± 0.09
ERM + SGAN, 0%	5.06 ± 0.36	3.21 ± 0.21
ERM + LGAN, 0%	4.82 ± 0.28	3.22 ± 0.27

ERM, 1%	4.27 ± 0.31	3.34 ± 0.25
**ERM + CSYNTH**, 1%	**3.51**± 0.25	**3.03**± 0.04
ERM + SGAN, 1%	4.08 ± 0.24	3.18 ± 0.18
ERM + LGAN, 1%	3.86 ± 0.30	3.20 ± 0.13

ERM, 10%	3.92 ± 0.35	3.01 ± 0.33
**ERM + CSYNTH**, 10%	**3.43**± 0.31	**2.91**± 0.05
ERM + SGAN, 10%	3.81 ± 0.27	2.98 ± 0.14
ERM + LGAN, 10%	3.72 ± 0.34	3.00 ± 0.21

ERM, 25%	3.80 ± 0.26	2.93 ± 0.04
**ERM + CSYNTH**, 25%	**2.92**± 0.11	**2.89**± 0.12
ERM + SGAN, 25%	3.74 ± 0.31	2.97 ± 0.11
ERM + LGAN, 25%	3.45 ± 0.28	2.91 ± 0.13

#### 3.2.3 Experiment: confounders

In Sections 3.2.1, 3.2.2 we demonstrated CounterSynth’s ability to rectify poor population and worst-subpopulation performance given a demographically imbalanced training set. In those experiments the demographic attribute was not correlated with the target label — in this section we examine what happens when it is.

A correlation between demographic and pathological labels is common in medical imaging. Many neurological disorders, such as neurovascular and neurodegenerative disorders, exhibit marked correlation with age (Arntz et al., 2016, Philpot et al., 1990); others, such as neuroinflammatory disorders, with sex (Spychala et al., 2017). A good example from UK Biobank imaging data is WMH volume: older participants tend to have higher WMH volumes (sample Pearson correlation coefficient of 0.38 with p<0.0005). Studies have highlighted possible associations between abnormally high WMH volumes and risks of stroke, cognitive decline and dementia (DeCarli et al., 1995, Debette and Markus, 2010).

**Fig. 9:**
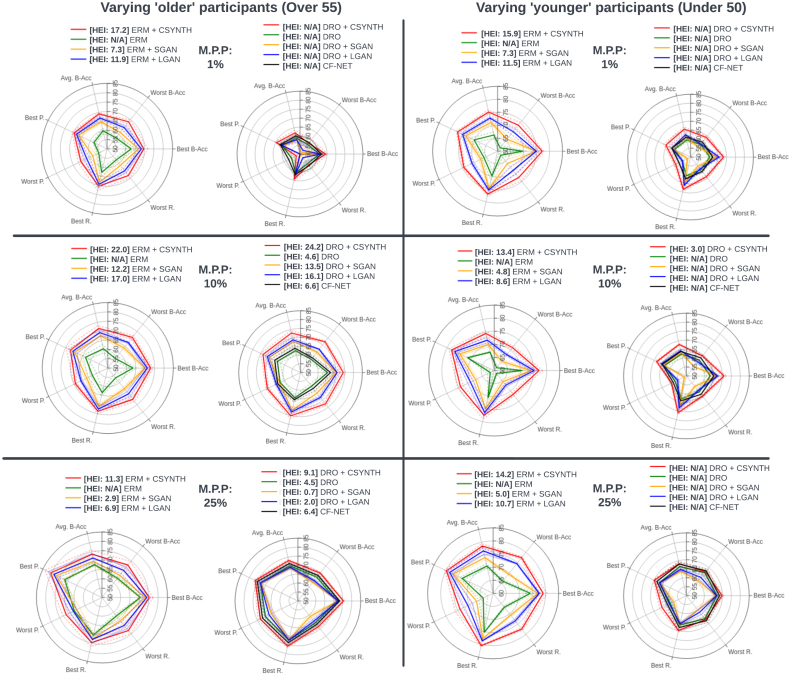
Spider plots depicting the performance of each model in terms of, on separate axes, average balanced accuracy (Avg B-Acc), best subpopulation balanced accuracy (Best B-Acc), worst subpopulation balanced accuracy (Worst B-Acc), best subpopulation precision (Best R.), worst subpopulation precision (Worst R), best subpopulation recall (Best R.), worst subpopulation recall (Worst R.) and, in the legend, the HEI score. The ideal model should be maximal along each axis, yielding an equilateral heptagon shape of maximum surface area, and should exhibit the largest HEI. Dotted lines indicate 1 standard deviation. The minority population percentage (M.P.P.) is manipulated across panels as indicated in the legend. *On the left*: Test set results for WMH volume classification with varying levels of imbalance for ‘older’ participants. The number of ‘young’ and ‘middle’ patients in the training and validation sets is 5153, 452 respectively. Of the ‘older’ participants in the training and validation sets respectively, 1% amounts to 52, 4 participants, 10% amounts to 572, 50 participants, and 25% amounts to 1717, 151 participants. *On the right*: Test set results for WMH volume classification with varying levels of imbalance for ‘younger’ participants. The number of ‘older’ patients in the training and validation sets is 6305, 566 respectively. Of the ‘younger’ participants in the training and validation sets respectively, 1% amounts to 64, 6 participants, 10% amounts to 700, 63 participants, and 25% amounts to 2101, 188 participants. Here ‘N/A’ indicates that ΔL≤0 (see Section 2.3), so the HEI does not apply.

In this section we simulate various age imbalances while training a classifier to predict whether a participant’s WHM volume is in the bottom three quartiles of the population versus the top quartile (see Section 2.6). We test the six approaches described at the start of Section 3.2 to counter the negative effects of this imbalance on performance and equity. In the first experiment the population defined by the demographic attribute most strongly correlated with the target, the ‘older’ participants, is under-represented in the training and validation data. The population defined by the demographic attribute least correlated with the target, the ‘younger’ participants, is over-represented. In the second experiment the converse is true. In both experiments we vary the ratio of both demographics. The results are presented in Fig. 9.

Fig. 9 shows that when the underrepresented population is negatively correlated with the target class (right side), ERM suffers a strong performance deterioration even when the representation percentage is high. On the other hand, for the positively correlated underrepresented population (left side), ERM does manage to partially correct the performance imbalances at 25% representation. Similarly to the experimental setup described in Section 3.2.1, both DRO and CF-Net fail to improve on ERM’s performance on the most underrepresented subpopulation when their representation is very low (1%). For higher subpopulation representations, Fig. 9 illustrates that the directionality of the correlation between the demographic and the target class is an important consideration to have when using both DRO and CF-Net. When the population positively correlated with the target is underrepresented the methods improve on ERM’s performance on the underrepresented subpopulation. However, in the reverse situation, both methods fail to lead to improved performance.

We again see across-the-board performance improvements when using counterfactual augmentation. Similarly to the experimental setups presented in the previous two sections, SGAN provides the smallest improvements, followed by LGAN and CounterSynth. The improvements obtained are very similar across cases, regardless of the directionality of correlation.

#### 3.2.4 Experiment: collider bias

Here we evaluate how our six approaches remedy performance and equity impaired by a collider bias (Griffith et al., 2020, Watson et al., 2019, Sperrin et al., 2016), where two demographic attributes are correlated with the target variable. Collider bias occurs when data collection is incidentally conditioned on a particular attribute, resulting in a distorted/false correlation between that attribute and a target variable.

We use the experimental setup from the previous section, where the target is WMH volume and one of the confounding demographic attributes is age. We do not evaluate CF-Net because the official implementation (Zhao et al., 2020) does not support multiple confounders.

Here we simulate a collider bias by conditioning the generation of two training and validation sets on sex. We begin with all of the ‘older’ males with top quartile WMH volume (see Section 2.6), then add participants, in equal proportions male, female, ‘younger’, ‘middle-aged’ and ‘older’, until they make up 1% of the total: this constitutes the first set. We then repeat the process, but now continuing to add participants until 10% and 25% of the total is reached. The test sets are, as always, sampled from the natural distribution.

In the 10% set the sample Pearson correlation coefficient between sex and the target is 0.84 with p<0.0005 and between age and the target it is 0.42 with p<0.0005. In the 25% set the sample Pearson correlation coefficient between sex and the target is 0.81 with p<0.0005 and between age and the target it is 0.39 with p<0.0005. For reference, the Pearson correlation on the natural UK Biobank distribution between sex and abnormally high WMH volumes is only 0.04 with p<0.0005.

**Fig. 10:**
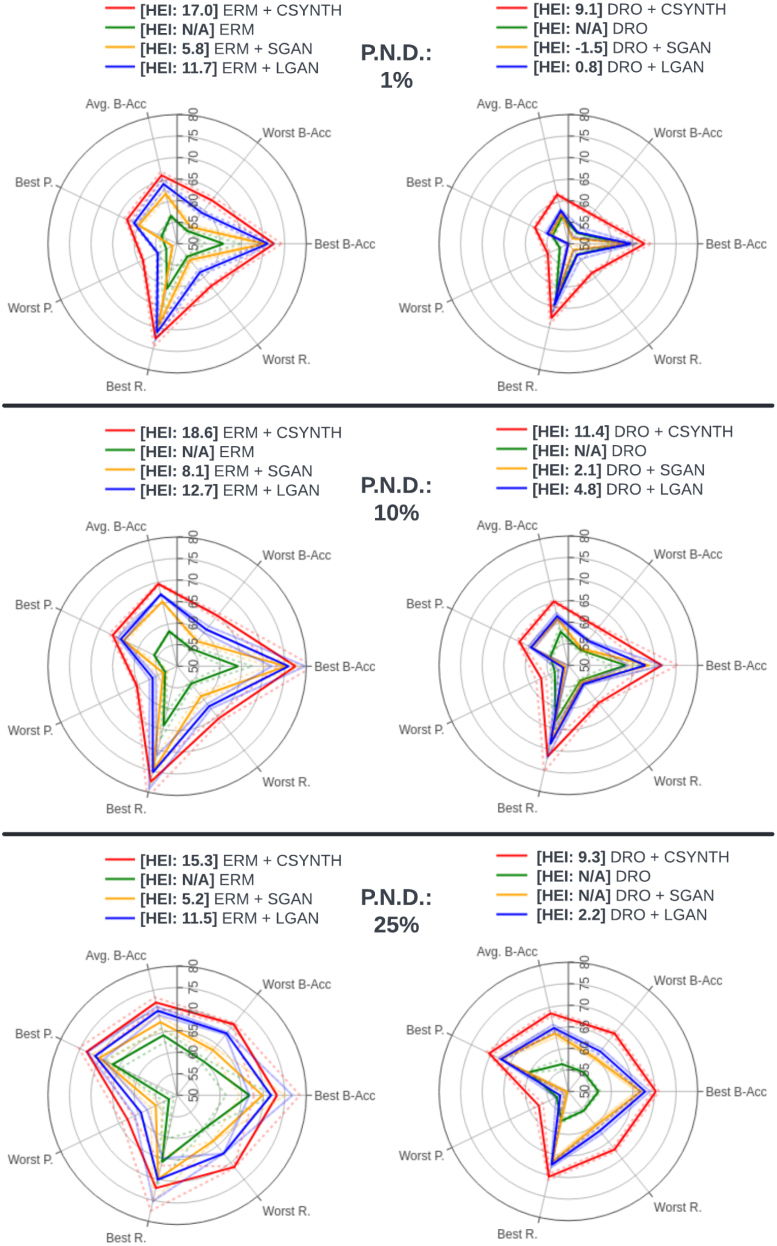
Spider plots depicting the performance of each model in terms of, on separate axes, average balanced accuracy (Avg B-Acc), best subpopulation balanced accuracy (Best B-Acc), worst subpopulation balanced accuracy (Worst B-Acc), best subpopulation precision (Best R.), worst subpopulation precision (Worst R), best subpopulation recall (Best R.), worst subpopulation recall (Worst R.) and, in the legend, the HEI score. The ideal model should be maximal along each axis, yielding an equilateral heptagon shape of maximum surface area, and should exhibit the largest HEI. Dotted lines indicate 1 standard deviation. The percentage of the natural distribution (P.N.D.) is manipulated across panels as indicated in the legend. Test set results for WMH volume classification with sex and age as collider variables. Here ‘N/A’ indicates that ΔL≤0 (see Section 2.3), so the HEI does not apply.

For each combination of model and collider bias we present (with standard deviation): (1) balanced accuracy; (2) precision and recall for the best- and worst-preforming subpopulation; (3) average balanced accuracy; and (4) our index, HEI. The results are summarised in Fig. 10.

Fig. 10 shows that collider bias leads to the worse overall ERM performances on the WMH task. Especially at lower representations (1% and 10%) of the natural distribution, the model suffers big performance hits, not only on the most underrepresented population but also on the entire test set. We notice that aside from when using counterfactuals, DRO methods fail to improve on ERMs performance on all cases. Our experimental setup includes a case not originally studied in Sagawa et al. (2020) and demonstrates the methods needs some restructuring in order to successfully operate under the effects of collider bias and distributional shift. This reported failure case for DRO is consistent with the findings of Taori et al. (2020).

When using data augmentation, similarly to the experimental setups presented in previous sections, SGAN provides the smallest improvements, followed by LGAN and CounterSynth. This demonstrates the usefulness of counterfactual data augmentation in optimising predictions obtained in situations such as clinical studies.

## 4 Discussion

With arguably the greatest strength of complex modelling – its individuating power – comes a critical vulnerability: differential performance across diverse subpopulations in proportion to their representation in the training data (Buolamwini and Gebru, 2018, Larrazabal et al., 2020, Hashimoto et al., 2018, Barocas et al., 2017). Where the sampling of the foreground signal of interest is insufficient to reveal its structure, to any conceivable model, adding more data is the only viable solution. But where differential performance arises from the conflation of foreground and incidentally correlated, irrelevant background features, systematic manipulation of the background alone may provide an adequate remedy. Crucially, such manipulation may be informed by data from another domain, executed by models trained under large-scale data regimes infeasible in the target domain.

Here we demonstrate for the first time in the realm of brain imaging a robust method for achieving this by counterfactual synthetic data augmentation constrained to morphological features of the background. We show that this approach can enhance performance on minority subpopulations defined by multiple interacting factors, promoting equity without a cost to the rest of the population, indeed with added benefit (Section 3.2). Whereas a closed framework, reliant on redistributing model attention within the domain, such as group distributionally robust optimisation (Sagawa et al., 2020), will generally improve performance in one subpopulation at the cost of degrading it in another, an open framework that transfers knowledge from another domain has the potential to improve equity at no overall cost.

Seven points of necessity, optimality, generalisability, and scope arise.

First, it should be recognised that in medicine the acquisition of large scale data is often limited by constitution rather than practicality. Neurology in particular is replete with pathological conditions too rare to allow the data scales to which contemporary machine vision architectures are accustomed. Amyotrophic Lateral Sclerosis, for example, is diagnosed in only 670 new patients across the UK annually. Operating with comparatively small scale data is, and always will be, the norm here, making data efficiency an essential aspect of complex analytic methods with real-world ambitions.

Second, if the necessity for conventional data augmentation, such as geometric transformations, is conceded by its widespread use in contemporary medical imaging models, then its extension to other features to which invariance should be promoted is entirely natural. Note that the biologically-informed augmentation introduced here cannot plausibly be replaced by random non-linear transformations that could superficially mimic it because to achieve adequate disentanglement from correlated factors we need to replicate biologically structured patterns of background variation.

Third, though non-linear image registration can be used to homogenise images morphologically (Klein et al., 2010), it does not provide a practicable means of reducing background contextual entanglement. The regularisation on which robust non-linear registration depends inevitably retains substantial morphological signals as demonstrated by the excellent performance of age regression and sex-classification models on registered data (Section 3.1). Moreover, whereas augmentation need only be confined to training, a registration-dependent analytic framework requires test data to be transformed into the same space: a task not easily accomplished without interference from foreground pathology.

Fourth, the proposed augmentation strategy does not assume, but is inevitably sensitive to, the preservation of the foreground signal in the act of translation. This is the core rationale for restricting the generator to diffeomorphic morphological deformations that leave tissue intensity signals broadly intact (Section 3.1.1). Where the foreground signal is itself morphologically conveyed, the synthetic mechanism may conceivably distort it. But whether or not such distortion offsets the benefits of augmentation is quantifiable at test time, and will depend on the task and the nature of the pathology. Crucially, the use of a more expressive synthetic model is not necessarily desirable, for the risk of distortion or even erasure of the pathological signal is thereby increased. In situations where the background requires an intensity-based manipulation, an analogous non-morphological generative architecture would be appropriate.

Fifth, though our method is here applied to the promotion of equitable model performance, its use has the potential to harden a model to distributional shift (Kaushik et al., 2020) and reduce the risk of underspecification (D’Amour et al., 2020, Larrazabal et al., 2020) by counterfactually exposing it to a wider diversity of plausible foreground-background combinations than the training data alone contains. This should not only lessen model dependence on domain-specific features with poor generalisability, but enable training a model to become cognisant of specific, directed, counterfactually-defined contextualising backgrounds, before they are even encountered in the wild.

Sixth, the ability to learn, transferrably, a characteristic such as age or sex from a set of data will be sensitive to other characteristics, such as the presence of incidental pathology, to the extent to which they interact with it. While we minimise this sensitivity by constraining the expressivity of our synthetic mechanism to modulations of morphology, its magnitude is an empirical question to be answered in any specific modelling scenario by quantifying the fidelity of retrieval of the conditioning characteristic from a separate set of synthesised data. Performing such quantification on the target set of interest may be complicated by the presence of pathology, but the value of the overall augmentation process is in any event ultimately determined by the fidelity of the downstream task, evaluated on held out data.

Finally, casting light on the equity of model performance across subpopulations reveals a pressing need for a quantitative ethical framework that allows formal comparison across both architectures and trained models. Here we build on concepts derived from econometrics to suggest a novel index (Section 2.3), the Holistic Equity Index, that addresses the specific needs of the task, with potential utility in other areas.

## 5 Conclusion

CounterSynth is a novel conditional generative model of diffeomorphic deformations that induces label-driven, biologically plausible changes in volumetric brain images with potential utility in enabling biologically structured counterfactual augmentation.

We demonstrate by longitudinal data ageing prediction (Section 3.1.3), voxel-based morphometry (Section 3.1.1), demographic classification (Section 3.1.4), and Fréchet inception distance (Section 3.1.2) that CounterSynth produces anatomical deformations closely replicating the actual demographic morphological differences observed in UK Biobank and OASIS-3 data.

Extensive comparative evaluation (Section 3.2) on demographically imbalanced tasks with and without confounders further demonstrates that the use of counterfactual augmentation results in state-of-the-art improvements to both overall fidelity and equity of discriminative models, optionally operating in synergy with other fairness methods such as DRO.

The enviable power of complex modelling in the realm of medical imaging has brought increased focus on the necessity to match performance with equity across heterogeneous populations. Our model and associated analyses cast light on the problem of equity in modelling brain images, and provide theoretical and practical elements of a framework that will enable researchers and clinicians to tackle it head on.

## Declaration of Competing Interest

The authors declare the following financial interests/personal relationships which may be considered as potential competing interests: Guilherme Pombo reports financial support was provided by Wellcome Trust. Guilherme Pombo reports financial support was provided by NIHR UCLH Biomedical Research Centre.

## Data Availability

The data used is open source (UK Biobank and OASIS) and is available to anyone who request it, but cannot be shared by the authors, as the request needs to go through the appropriate entities.

## Footnotes

1 Each evaluation of the cycle loss (2) requires two forward passes through the generator, one of which is required to evaluate (1) the other we now avoid.

2 The extent to which this necessitates affine as opposed to just rigid registration of test data depends on how much spatial augmentation is applied during training; see Section 2.4. Affine registration is not a *necessary* part of our method: it simply discourages CounterSynth from learning trivial deformations, such as the shrinking brain size associated with ageing. Alternatively this can be achieved through more aggressive spatial augmentations during training, but naturally this slows convergence.
